# Who pays for healthcare in Bangladesh? An analysis of progressivity in health systems financing

**DOI:** 10.1186/s12939-017-0654-3

**Published:** 2017-09-06

**Authors:** Azaher Ali Molla, Chunhuei Chi

**Affiliations:** 1 Institute of Health Economics, University of Dhaka, Dhaka, Bangladesh; 2 Department of Applied Health Sciences, Public and Community Health, School of Nursing and Health Professions, Murray State University, Murray, KY USA; 3 School of Biological and Population Health Sciences, Milam 13, Corvallis, OR 97331-5109 USA; 4 Graduate Program in Health Management and Policy, College of Public Health and Human Sciences, Oregon State University, Milam 13, Corvallis, OR 97331-5109 USA; 5 Graduate Program in Applied Economics, Oregon State University, Milam 13, Corvallis, OR 97331-5109 USA; 6 Graduate Program in Public Policy, Oregon State University, Milam 13, Corvallis, OR 97331-5109 USA

**Keywords:** Progressivity, Ability-to-pay, Household healthcare expenditure, Health equity, Out-of-pocket payments

## Abstract

**Background:**

The relationship between payments towards healthcare and ability to pay is a measure of financial fairness. Analysis of progressivity is important from an equity perspective as well as for macroeconomic and political analysis of healthcare systems. Bangladesh health systems financing is characterized by high out-of-pocket payments (63.3%), which is increasing. Hence, we aimed to see who pays what part of this high out-of-pocket expenditure. To our knowledge, this was the first progressivity analysis of health systems financing in Bangladesh.

**Methods:**

We used data from Bangladesh Household Income and Expenditure Survey, 2010. This was a cross sectional and nationally representative sample of 12,240 households consisting of 55,580 individuals. For quantification of progressivity, we adopted the ‘ability-to-pay’ principle developed by O’Donnell, van Doorslaer, Wagstaff, and Lindelow (2008). We used the Kakwani index to measure the magnitude of progressivity.

**Results:**

Health systems financing in Bangladesh is regressive. Inequality increases due to healthcare payments. The differences between the Gini coefficient and the Kakwani index for all sources of finance are negative, which indicates regressivity, and that financing is more concentrated among the poor. Income inequality increases due to high out-of-pocket payments. The increase in income inequality caused by out-of-pocket payments is 89% due to negative vertical effect and 11% due to horizontal inequity.

**Conclusions:**

Our findings add substantial evidence of health systems financing impact on inequitable financial burden of healthcare and income. The heavy reliance on out-of-pocket payments may affect household living standards. If the government and people of Bangladesh are concerned about equitable financing burden, our study suggests that Bangladesh needs to reform the health systems financing scheme.

## Background

The relationship between payments toward healthcare and ability to pay (ATP) is a common measure of equity [1]. Who pays how much share for healthcare is a question of financial fairness. The measurement of progressivity or regressivity is important not only for a wide range of equity perspective, but also for macroeconomic and political analysis of healthcare systems [2]. Inequalities exist in almost all sectors but inequalities in the health sector have more negative impacts than in other sectors [3, 4].

The issue of equity is widely acknowledged by health economists and health policy researchers to be an important policy objective in the healthcare field [5–8]. Its importance is recognized not only in low and middle-income countries (LMICs), but also in high-income countries (HICs). In Organization of Economic Cooperation and Development (OECD) countries, equity appears to be a prominent issue in the continuing debate on healthcare financing and delivery [9, 10]. In the same way, there is a strong agreement among the policy makers in low-income countries that equity should feature prominently in health policy decisions [11–13]. The WHO has placed health equity as the second of its thirty-eight targets within the new “Policy for Health for All”. The policy says that by the year 2020 the health gap between socioeconomic groups within countries should be reduced by at least one fourth in all member states [14].

Without a well-functioning healthcare financing system, timely access to health services cannot be achieved for the majority of the population. The system itself determines whether people can afford to use health services without any financial hardships when they need them. Thus, recognizing the importance of equitable health systems financing (HSF), the World Health Organization (WHO) committed and recommended that countries develop a financing system so that all people have access to services and do not suffer financial hardship paying for them [14]. The sixty-fourth World Health Assembly [15] urged member states to avoid significant direct payments at the point of delivery, and to include a method for prepayment of financial contributions for healthcare and services. It also urged members to devise a mechanism to pool risks among the population to avoid catastrophic healthcare expenditure and impoverishment of individuals and households.

With an area of 56,977 miles and a population of 161.03 million [16], Bangladesh is going through a demographic and epidemiologic transition [17]. The country is the eighth most populous country of the world while it is the 94th largest by area. 28.27% of the populations are below 14 years of age and 6.04% are above 65 years with a dependency ration 52.5%. The population growth rate is 1.05% (2016 est.) with a life expectancy at birth of 73.2 years. Yet food and waterborne diseases (bacterial and protozoal diarrhea, hepatitis A and E, and typhoid fever) and vector-borne diseases (dengue, malaria) are highly prevalent. The economy has grown roughly 6% per year since1996 [18].

Bangladesh HSF is characterized by increasingly high out-of-pocket (OOP) payments, and, at the same time, the absence of an active prepayment system [18]. In Bangladesh, OOP payments as percent of private expenditure on health (92.9%) is higher than India (89.2%) and Nepal (79.9%) [19]. The Bangladesh National Health Accounts [18] reports that household OOP expenditure remains the main source of HSF, increasing from 56.9% in 1997 to 63.3% in 2012 of total health expenditure (THE). The second largest financing agent is government, making up 26.0% of THE. The private firms’ share has remained at around 1.0% over the years. The share of non-government organizations (NGOs) from their own sources has ranged between 1% and 2.0% of the THE over the 1997–2012 period. Development partners contribute through NGOs or government. The rest of the expenditure through NGOs varied from 5% to 9% during the period. Household expenditure as a percentage of GDP increased from 1.6% to around 20.0% in 2010.

The health spending in Bangladesh accounted for 3.4% of gross domestic product (GDP), which is lower than the average (3.8%) in South East Asia (SEA) region, below the average of low-income countries (5.4%), lower-middle income countries (4.3%), and far below the world (8.5%) [19]. A breakdown of OOP expenditure shows that drugs and medicine constitute 65.0% of OOP spending. Other components of OOP are services of curative care (22.0%), ancillary services (9.0%), outpatient and home-based services (4.0%), and general government administration of health (less than 1.0%).

The consequences of excess OOP expending are enormous with different scenarios. Some households may not utilize formal healthcare at all due to excess OOP payments, or they may receive partial care and thus aggravate the disease condition, causing the disease to become a chronic condition. Households may sell their movable and immovable properties to manage the treatment costs, which in turn make them poorer. Due to excess health expenditures, households may need to ration their food items, and thus may become malnourished. OOP health expenditure may affect education, causing children to drop out of school. Moreover, OOP payments may mislead planners and policy makers to miscalculate poverty status.

Policy makers and planners are not fully aware of the situation due to the acute shortage of health policy research in Bangladesh. We aim to analyze progressivity to examine if inequities in health systems financing (HSF) exist. This will provide an evidence base for health planners and policy makers who want to promote equity in HSF. In addition to numerical differences, this study will employ statistical and macro-economic analysis and techniques to examine equity using Bangladesh Household Income and Expenditure Surveys [20]. This research seems to be the first of its kind to analyze of progressivity of HSF in Bangladesh using nationwide survey dataset.

We evaluated progressivity of HSF from all sources of available financing: tax, social insurance, private insurance, and OOP payments. We did not consider foreign aid, as it is not relevant because our purpose was to evaluate the distributional impact of domestic source of health systems financing on the domestic population. Assuming tax parameters have been set for foreign loan repayment, the distributional burden on the current generation of foreign debt financing will be captured through the evaluation of the tax distribution.

## Data and methods

### Sampling technique

We use the Bangladesh Household Income and Expenditure Survey [20] dataset conducted by Bangladesh Bureau of Statistics (BBS). This is the source of data for estimating household income, expenditure, consumption, income inequality and incidence of poverty. For this round, data collection was started on 1st February 2010 and continued up to 31st January 2011.

A two-stage stratified random sampling technique was followed in drawing the sample for this survey under the framework of Integrated Multipurpose Sampling (IMPS) design developed on the basis of the 2001 Bangladesh population and housing census. In IMPS design, the whole country was divided into 16 strata, which included six from rural, six from urban, and four from sub-municipal areas (SMAs). The design consists of 1000 primary sampling units (PSUs) throughout the country systematically drawn from the 16 strata. Out of 1000 PSUs, 640 were from rural and 360 from urban areas. Each PSU is comprised of approximately 200 households. In the first stage, 612 PSUs were drawn from 1000 PSUs. In the second stage, 20 households were randomly selected from each PSU. Thus, PSUs selected for HIES 2010 are actually a subset of PSUs of the IMPS design. The total sample size stands at 12,240 households comprising of a population of 55,580.

## Methods

We used **t**wo methods to measure the progressivity of health payments: comparing the share of health payments to their share of ATP, and assessing departure from proportionality or Lorenz dominance analysis. Under the progressive system, the share of health payments are less than their ATP and the Lorenz curve dominates (lies above) the concentration curve. The opposite is true for a regressive system. Kakwani index is being used to measure the magnitude of progressivity.

We have used STATA 14.0 [21] and the Automated Development Economics and Poverty Tables (ADePT) software, version 5.0 developed by World Bank’s experts [22]. Progressivity is assessed using a direct and a less direct method. A direct method is a percentage of OOP payments for healthcare as a percentage of total household expenditure by quintile/decile groups of equivalent household expenditure. A less direct method of assessing progressivity is defined in relation to departure from proportionality. This method compares the share of health payments contributed by proportions of the populations ranked by ATP with their share of ATP. It compares the concentration curve of health payments (LH_(p)_) with the Lorenz curve for ATP, (L_(P)_). The merit of this curve is that it provides a visual representation of the distribution information. However, it does not show the distribution exactly, and it is difficult to compare this curve between the countries.

Because the Lorenz dominance analysis alone does not provide a measure of magnitude of proportionality, Kakwani index [23] is used to measure the magnitudes of progressivity/regressivity. Kakwani index is twice the area between a payment concentration curve and a Lorenz curve. It is calculated as

1 $$ {\pi}_k=C-G $$

Where, *C* is the concentration curve and *G* is the Gini coefficient of the ATP variable. The Gini coefficient (*G*) is used to measure of inequality of a distribution. The value of *G* varies from 0 to 1. The Gini coefficient is regarded as the gold standard in economic analysis in assessing inequality.

### Key variables

The variables for this part of analysis are ability to pay (ATP) (Table 1), food consumption, non-food consumption, and amount of healthcare payments. Ability to pay for each household was calculated by adding all forms of consumption such as food consumption and non-food consumption. The amount of healthcare payments was calculated by adding all the related costs of healthcare including direct and indirect costs.

**Table 1:** Variables with definitions and source

Variables	Definitions	Source
Ability to pay (ATP)	Households’ yearly consumptions of food, non-food and payments towards healthcare^a^	HIES, 2010
Food consumption	Market price of food items consumed in one year^a^	HIES, 2010
Nonfood consumption	Market price of nonfood items in one year^a^	HIES, 2010
Healthcare payments	Gross of all payments towards healthcare, including direct tax, indirect tax, social insurance and private insurance^a^	HIES, 2010

^a^Measured in Bangladesh currency Taka (Tk.). 1 Tk. = US$ 0.08

## Results

The summary statistics (Table 2) show that out of 12,240 households, 11,638 households were included for analysis. The mean household size is 4.5 persons, ranging from 1 to 17 members per household. The mean households’ aggregate annual consumption is Tk. 132,510 (US$ 1656) with a range from a minimum of Tk. 15,327 (US$191.6) to a maximum of Tk. 1,843,160 (US$23,039). Nonfood consumption varies from Tk.4827 (US $60) to Tk.1,712,261 (US $21,403). There are more observations below the mean (right skewed) for both total consumption and nonfood consumption. The average contribution of tax is Tk.754, social health insurance is Tk. 142 US $9.4), and private health insurance is Tk.8 (US $0.1). Total OOP payments range from zero to Tk.1,369,000 (US $17,113) with an average of Tk.5339 (US $76) and a median of Tk.2200 (Us $28). Majority of observations are below the mean.

**Table 2:** Summary report of sample data (HIES), Bangladesh 2010

	N	mean	min	max	p1	p50	p99	N_unique
Household size (person)	11,638	4.5	1.0	17.0	1.0	4.0	10.0	17
Total HH consumption (Tk.)	11,638	132,510	15,327	1,843,160	40,001	110,443	474,890	11,632
Total NF consumption (Tk.)	11,638	62,071	4827	1,712,261	10,387	42,050	354,297	10,654
Household sampling weights	11,638	2739	897.1	6882.3	897.1	2946	6882.3	16
Household tax (Tk.)	11,638	754	0.0	200,000	0.0	0.0	20,000	184
Social health insurance (Tk.)	11,638	142	0.0	120,000	0.0	0.0	3000	76
Private health insurance (Tk.)	11,638	8	0.0	50,000	0.0	0.0	0.0	9
Total OOP expenditure (Tk.)	11,638	5339	0.0	1,369,000	0.0	2200	50,000	1594

Note: p1 = 1st percentile, p50 = 50th percentile, p99 = 99th percentile

### Health systems financing

The average per capita consumption for lowest, second and third quintile is lower than the total average (Tk.29,907), which indicates that majority of the population consumes less than the average (Table 3). Total consumption in the poorest quintile (Tk. 13,529) is less than half (Tk. 29,908) of the total average consumption. Direct tax appears to be born significantly higher by the richest quintile. It is also important to note that the amount of social insurance is very small. Contribution to private health insurance for each and every quintile is very negligible and insignificant. Out-of-pocket expenditure constitutes a major portion of health finance. The poorest quintile contributes Tk.842, which is half of the richest quintile (Tk.1594). Per capita consumption gross for the poorest quintile is Tk.13,529 and per capita consumption net of healthcare payments is Tk.12,635. Households in the poorest quintile consume 0.22 times the richest quintile in respect of gross consumption and 0.21 times in respect of net consumption. This clearly shows an inequity in healthcare payments between the poorest and the richest quintiles.

**Table 3:** Average Per Capita Health Finance by quintiles, Bangladesh 2010

	Per capita consumption, gross (Tk.)	Household yearly tax in (Tk.)	Household yearly social health insurance in (Tk.)	Household yearly private health insurance in (Tk.)	Household yearly out-of-pocket health expenditure in (Tk.)	Total payments in (Tk.)	Per capita consumption, net of payments in (Tk.)
Lowest quintile	13,529	49	36	0.0	842	927	12,635
Standard error	62	9	9	0.0	43	47	73
2nd quintile	19,076	75	36	2	1114	1226	17,940
Standard error	34	15	8	1	89	92	63
3rd quintile	24,032	91	52	7	1378	1528	22,705
Standard error	40	15	16	5	171	173	69
4th quintile	31,690	107	63	0.0	1331	1500	30,271
Standard error	73	20	19	0.0	95	100	97
Highest quintile	61,204	192	58	0.2	1594	1844	59,424
Standard error	759	36	22	0.25	81	93	760
Total	29,907	103	49	1.8	1252	1405	28,596
standard error	238	9	7	0.93	47	49	238

*Note: 1 Bangladesh Taka (Tk.) equals 0.08 dollars*

### Progressivity or regressivity?

Tables 4 and 5 analyze the progressivity of HSF. Table 4 gives the average consumption and financing share by quintile, with households ranked in ascending order of gross consumption. Information related to gross consumption gives an idea about income inequality; the greater of the richest quintiles, the greater the inequality.

**Table 4:** Household Share of Total Healthcare Financing, Bangladesh 2010

	Per capita consumption, gross (%)	Household yearly tax (%)	Household yearly social health insurance (%)	Household yearly private health insurance (%)	Household yearly out-of-pocket health expenditure (%)	Total payments %	Per capita consumption, net of payments (%)
Quintiles of per capita consumption, gross							
Lowest quintile	9.0	9.5	14.8	0.0	13.4	13.2	8.8
standard error	0.25	1.83	3.72	0.00	0.83	0.79	0.24
2	12.8	14.6	14.7	17.8	17.8	17.5	12.5
standard error	0.32	2.78	3.38	13.19	1.37	1.27	0.32
3	16.1	17.7	21.3	79.4	22.0	21.8	15.9
standard error	0.39	2.91	5.95	14.19	2.23	2.04	0.39
4	21.2	20.9	25.5	0.0	21.3	21.4	21.2
standard error	0.48	3.52	6.55	0.00	1.45	1.37	0.49
Highest quintile	40.9	37.4	23.7	2.8	25.5	26.2	41.6
standard error	0.72	4.96	7.43	3.11	1.37	1.35	0.73
Total	100.0	100.0	100.0	100.0	100.0	100.0	100.0
standard error	0.00	0.00	0.00	0.00	0.00	0.00	0.00
Gini coefficient	0.3134						0.3276
standard error	0.00						0.00
Concentration Index		0.2419	0.1040	−0.1208	0.1128	0.1217	
standard error		0.05	0.07	0.06	0.02	0.02	
Kakwani index		−0.0714	−0.2094	−0.4342	−0.2005	−0.1917	
standard error		0.05	0.07	0.06	0.02	0.02

**Table 5:** Financing Budget Shares, Bangladesh 2010 (Health Financing as a Share of total gross consumption)

	Per capita consumption, gross	Household yearly tax	Household yearly social health insurance	Household yearly private health insurance	Household yearly out-of-pocket health expenditure	Total payments	Per capita consumption, net of payments
Quintiles of per capita consumption, gross							
Lowest quintile (%)	100.0	0.4	0.3	0.0	6.2	6.8	93.4
standard error	0.00	0.07	0.07	0.00	0.32	0.34	0.29
2nd quintile (%)	100.0	0.4	0.2	0.0	5.8	6.4	94.0
standard error	0.00	0.08	0.04	0.01	0.47	0.48	0.28
3rd quintile (%)	100.0	0.4	0.2	0.0	5.7	6.4	94.5
standard error	0.00	0.06	0.07	0.02	0.71	0.72	0.24
4th quintile (%)	100.0	0.3	0.2	0.0	4.2	4.7	95.5
standard error	0.00	0.06	0.06	0.00	0.30	0.31	0.20
Highest quintile (%)	100.0	0.3	0.1	0.0	2.6	3.0	97.1
standard error	0.00	0.06	0.04	0.00	0.14	0.16	0.12
Total (%)	100.0	0.3	0.2	0.0	4.2	4.7	95.6
standard error	0.00	0.03	0.02	0.00	0.16	0.17	0.09
Gini coefficient	0.3134						0.3276
standard error	0.00						0.00
Concentration Index		0.2419	0.1040	−0.1208	0.1128	0.1217	
standard error		0.05	0.07	0.06	0.02	0.02	
Kakwani index		−0.0714	−0.2094	−0.4342	−0.2005	−0.1917	
standard error		0.05	0.07	0.06	0.02	0.02	

The poorest quintile consumes, on average, 9.0% of total (gross) consumption, whereas this amounts to 41.0% for the richest quintile (Table 4). Taxes appear to be borne mostly by the upper three quintiles, 17.7, 20.9 and 37.4%. The lowest two quintiles make up 9.5 and 14.6% of the total. The financing share increases by quintile for taxes. In case of social health insurance, the fourth quintile paid the most (25.5%) followed by the highest quintile (23.7%). The lowest and the second quintile make up nearly the same percent for social insurance (14.8 and 14.7%). In both per capita gross consumption and household yearly tax, the richest quintile bears the greater share, 40.9 and 37.4% respectively, which are 4.5 and 4.0 times respectively higher than the lowest quintile. In respect of social health insurance, the poorest quintile bears a 14.8% share, whereas the richest quintile bears 23.7%.

Private health insurance is either absent, or is present only in some pocket areas. The middle class or third quintile possesses the highest share of private health insurance (79.4%). Whereas, the poorest and the fourth quintiles have no private insurance and the richest quintile has only a 2.8% share.

Financing share for household yearly OOP expenditure for the poorest quintile is approximately half (13.4%) of the richest quintile (25.5%).

The discrepancies between the share of gross consumption and OOP payments are clearly visible. The comparison between per capita gross consumption and per capita consumption net of health payments shows that share decreases among the poor (9.0 vs 8.8%) and increases among the rich (40.9 vs. 41.6%), which indicates that post-healthcare payments consumption decreases among the poor and increases among the rich. This finding supports figures for the Gini coefficient (Table 5); prepayment Gini or income inequality is less than the post-payment Gini or income inequality (0.3134 vs. 0.3276). The concentration indexes are positive except households’ private health insurance. This indicates the wealthier contribute more in absolute amount to the financing of healthcare than the poor do. For the private health insurance, the concentration index is negative which is an indication of a regressive mode of financing. The concentration index is largest for household yearly taxes (0.2419) suggesting that taxes are relatively progressive than other sources. The differences between Gini coefficient of per capita gross consumption and concentration index or Kakwani Index for all sources of healthcare financing are negative. This indicates regressivity, meaning that OOP payment is more concentrated among the poor (Table 5).

Table 5 presents health financing as a share of total gross consumption. Household OOP healthcare expenditure remains the highest share (4.2%) of all healthcare financing (tax, social insurance and private insurance). The lowest quintile spends 6.8% of their total gross consumption for healthcare compared to 3.0% for the highest quintile. This clearly shows a regressive mode of financing. The contribution of household yearly private insurance tends to zero for nearly all the quintiles. Household yearly tax and social insurance are minimal, consisting of 0.3 and 0.2% respectively.

**Fig. 1:**
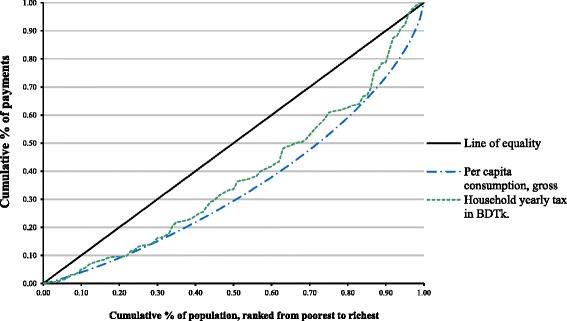
Lorenz dominance analysis of household tax, Bangladesh2010

**Fig. 2:**
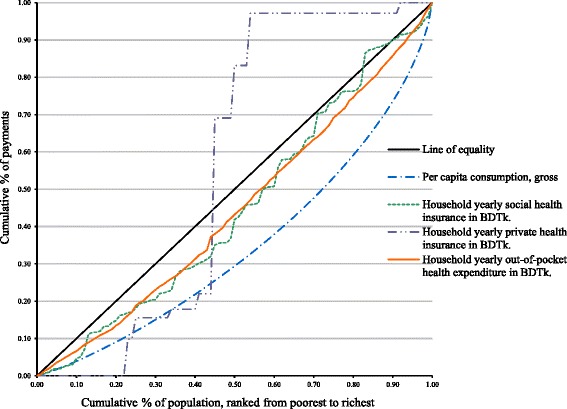
Lorenz dominance for sources of health system finance, Bangladesh 2010

Figure 1 shows the concentration curve for household taxes. The concentration curve of household tax lies inside the Lorenz curve at all levels of consumption. This suggests regressivity, which means that the poor pay proportionately more of their total household consumption for healthcare than the rich. In Fig. 2, both OOP expenditure and social insurance lie inside the per capita consumption gross (Lorenz curve), which indicates regressivity. Again, private insurance shows an abnormal peak just after the 20% mark of the population ranked from poorest to richest. As we discussed earlier, private insurance in Bangladesh exists in pocket areas of the country.

**Fig. 3:**
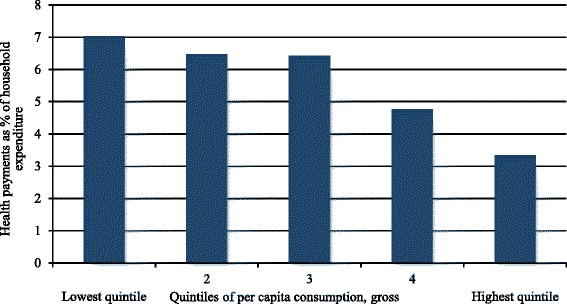
Health Payments shares by quintiles, Bangladesh, 2010

Finally, using the direct method, we have analyzed the overall progressivity (Fig. 3). It is the direct representation of the progressivity of health payments. It shows the health payments share by quintile. In Bangladesh, the share of health payments to household economic status, in our case consumption, decreases from the lowest quintile to the highest quintile. As is visible, the lowest quintile households pay 7.0% of their total consumption for healthcare, whereas, the highest quintile households pay about 3.3%. The bars show a sharp decreasing trend from lowest to highest quintile. We conclude that the HSF in Bangladesh is definitely regressive.

## Discussions

Our findings show that health system financing in Bangladesh is regressive in nature. Payments toward healthcare are not related to ATP. Healthcare payments account for decreasing proportion of ATP. As shown by the negative Kakwani indices, health systems financing is concentrated among the poor. This indicates that inequality exists in health systems financing. Our results confirm the conclusion of previous studies conducted by Wagstaff [24, 25], Van Doorslaer [26], and Mastilica [27] that OOP payments are a regressive means of healthcare revenue collection.

A limited number of studies from low-income countries exist, but there are no studies from Bangladesh. In our analysis, the wide range of OOP expenditure indicates a large gap between households in the lowest and the highest quintiles. Household yearly social insurance is minimal with mean of Tk. 142 and median of zero indicating that although social insurance exists it is limited within a group of households. Social insurance exists in formal sectors of Bangladesh. This explains why 3rd, 4th and 5th quintiles altogether bear 70.5% (Table 4, column 4) of financing altogether. Private health insurance is nearly absent existing only in some pocket areas. Traditional insurance markets are almost entirely absent in the rural areas of Bangladesh [28]. However, the findings show that the 3rd quintile bears the most financial burden of private insurance (79.4%). The poor cannot afford private insurance and the rich do not bother at all. Some NGOs initiated certain kinds of health insurance for the rural population. Grameen Bank also started to provide a micro health insurance scheme in the late 1990s. Similarly, Gonoshasthaya Kendra (GK) initiated some kind of health insurance in limited areas for the rural poor and middle class [28].

An inequality exists in per capita gross consumption in Bangladesh. On average, the lowest quintile consumes 0.22 times the highest quintile. Contrary to this, the rate of tax borne by the lowest quintile is 0.25 times that of the highest quintile. In respect to social health insurance, the lowest quintile bears 0.62 times what the highest quintile does.

The greater share of healthcare financial burden in household is OOP payments. The poor bear 0.53 times of the rich’s burden, whereas their consumption or ATP is only 0.22 of the rich. Proportionately, the poor pay more in OOP expenditures than the rich do. This makes the payments regressive or pro-rich.

It is clearly evident that the rich consume more than their poor counterparts, but at the same time, the rich pay proportionately less in taxes, social insurance, private insurance and OOP payments. For the poor, per capita net consumption decreases (9.0 vs. 8.8%) after OOP payments, whereas for the rich per capita net consumption increases (40.9 vs. 41.6%) after OOP payments (Table 2). This is related to the transfer payments and/or redistribution of disposable income, as we have seen in decomposition of redistributive effects. These findings are concordant with the Gini coefficients we estimated; prepayment Gini is less than the post payment Gini (0.3134 vs 0.3276). The positive concentration indexes indicate that the rich pay more in absolute terms but proportionately less. The concentration index is the largest for household yearly tax, suggesting that in terms of progressivity, taxes are relatively better than all other financing sources. Similarly, the Gini coefficient of per capita gross consumption and concentration index or Kakwani index for all sources of financing are negative; this indicates regressivity.

In Bangladesh, like other low-income countries, OOP payments contribute the greatest share of revenue (63.3%). The Lorenz dominance analysis indicates that the inequality exists in all sources of health systems financing (Figs. 1 and 2). Both the graphs offer a powerful means of representing the effect of health systems financing on the distribution of household living standards. It should be noted that this kind of analysis does not consider utilization of healthcare. Progressivity should not be interpreted as the rich paying more for the same amount of healthcare, as this is most often not the case and not accounted for the measure presented here.

The overall progressivity/regressivity analysis we performed used a direct measure. The results indicate, with little ambiguity, that it is regressive, as the lower income quintiles’ share of household consumption decreases with healthcare consumption. This clearly indicates that the health systems financing in Bangladesh is regressive.

The findings, although rich with both statistical and policy significance, should be interpreted in view of several limitations. Firstly, estimates of OOP payments from survey data are potentially subjected to both recall bias and small sample bias owing to the infrequency with which some healthcare payments are made. Secondly, our conventional measures of progressivity provide no information on those citizens who cannot afford to use health services and have incurred no health expenditures. Thirdly, the present study does not examine the obvious impact of OOP payments on the quantity and quality of care consumed. Our interpretations, therefore, need to be complemented by studies of health care utilization [29] and the incidence of public finance [30]. Finally, our analysis does not capture all potentially catastrophic effects of illness or disability, such as lost earnings, and does not investigate whether health shocks are absorbed by incurring debt or expending savings to smooth consumption [31].

## Conclusions

Our findings substantially add to the evidence on the regressivity of HSF in Bangladesh. This macro-level data analysis shows that there is heavy reliance on OOP payments, which exceed 63.0% of the total health expenditure. Further, this heavy reliance on OOP payments reduces household living standards and may lead to poverty or ultimately push households to deeper poverty.

Regressivity of Bangladesh HSF is mostly related to the high OOP and the absence of a functional collective prepayment system. Social insurance is very negligible, and private health insurance is present only in some pocket areas run by NGOs. The system urgently needs to introduce health insurance schemes for the poor, elderly, disabled and disadvantaged. Measurable country-specific milestones in monitoring progressivity need to be adopted. The findings of this study can guide policy decisions in this aspect.

Chronic illnesses and household income were found to be the most influential and statistically significant (*p* < 0.001) predictors of high household healthcare expenditure in Bangladesh [32]. Further, low revenue generating ability of the government of Bangladesh and high OOP expenditures make a risk pooling and prepayment system an absolute necessity. The Bangladesh Health Care Financing Strategy (HCFS), 2012–2032 recommends a national health protection scheme [33]. The scheme targets mainly the formal sectors of the country with compulsory payroll taxation. People below the poverty line would be subsidized from the general revenue. It would allow the informal sector to join the scheme voluntarily. Health cards have some positive effect on OOP payments [34]. Thailand introduced an identity card named “Health Card” in 1983 to protect the poor in the community [35]. Australian Health Care Card [36] and Indonesian Health Card Program [37] were introduced for the same purpose. Development of such type of health card may be an option for financial protection of the poor in Bangladesh.

The study findings may contribute to policy making, particularly in relation to the proposed financial risk protection, social protection, and universal coverage. Our findings provide empirical evidence for future healthcare reforms. We hope this research will stimulate more studies of this subject with improved method and analysis.
